# Screening essential oils for their antimicrobial activities against the foodborne pathogenic bacteria *Escherichia coli* and *Staphylococcus aureus*

**DOI:** 10.1016/j.heliyon.2019.e01860

**Published:** 2019-06-04

**Authors:** Julian Thielmann, Peter Muranyi, Pamina Kazman

**Affiliations:** a Technical University of Munich TUM, Munich, Germany; b Fraunhofer Institute for Process Engineering and Packaging IVV, Freising, Germany

**Keywords:** Microbiology

## Abstract

The application of essential oils as antimicrobials is a current subject of research and a promising approach in terms of natural food preservation. Due to the diversity of EO producing plant genera and the inconsistent use of susceptibility testing methods, information on the antibacterial potency of many EO varieties is fragmentary. This study was performed to assess the minimal inhibitory concentrations (MIC) of 179 EO samples from 86 plant varieties, using a single method approach, excluding emulsifying agents. MICs were acquired in a broth microdilution assay, using a dispersion based approach to incorporate EOs in a concentration range of 6400 to 50 μg/ml. *Staphylococcus aureus* and *Escherichia coli* were used as model bacteria. At concentrations below 400 μg/ml *S. aureus* was inhibited by 30, *E. coli* by 12 EO varieties. *Azadirachta indica* (50 μg/ml vs. *S. aureus*) *and Litsea cubeba* (50 μg/ml vs. *S. aureus*, 200 μg/ml vs. *E. coli*) essential oils were identified as promising new antimicrobial EO candidates with significant antimicrobial activity against the two foodborne pathogenic bacteria.

## 1 Introduction

Investigating the antimicrobial activities of plant essential oils (EO) has concerned many scientific studies within the last two decades. Besides a few general screenings [1, 2, 3], most studies were focused on one type of essential oil only, mainly *Thymus vulgaris, Origanum vulgare* or *Cinnamomum* species. Significant activities of these and other EOs against certain foodborne pathogenic bacteria such as *Escherichia coli, Listeria monocytogenes,* and *Salmonella typhimurium* have been demonstrated [4, 5, 6]. Furthermore, there has been continuative work regarding the identification of single active compounds from well investigated EOs, such as thymol, carvacrol or eugenol and partial elucidation of their cellular mechanism of action [7, 8, 9]. Most work on antimicrobial EOs was inspired by the idea of identifying alternative preservative agents with an overall “green” and “natural” or “bio-based” character for modern food technology applications.

The findings published to date, are still very fragmentary regarding a wide variety of essential oils [10]. Some EOs are still untested or negative results remain unpublished. In addition, it is difficult to reliably compare results from literature data, due to the strong variance of the used antimicrobial susceptibility testing methods. This complicates the selection of the most suitable EO candidates for further antimicrobial research and applications. Additionally, there are some constraints for EOs concerning food application, especially their sensory impact. EO components are considered aroma compounds (e.g. Thymol, Citral, Limonene, α-/β-Pinene, etc.), meaning they generally induce sensory activity [10]. Regarding food application it is therefore recommended to choose an essential oil based on the sensory profile of the targeted food product [11]. EOs with low inhibitory concentrations are considered advantageous to achieve antimicrobial effects in the product without affecting the sensory properties. For this study we defined a critical minimal inhibitory concentration (MIC) of 400 μg/ml *in vitro,* to select EOs with the most promising suitability for future application trials*.*

This study was performed to extent the available information on antibacterial activities of plant essential oils, intending to normalize the discussion on the antibacterial activity of plant essential oils with quantitative data for a large number of EOs. The comparability of the results is maximized by the use of a reproducible, quantitative method for MIC determination, combining the advantages of a dispersion approach, as developed by Remmal, Bouchikhi [12] and Friedman, Henika [13], with the reliability of a standardized broth microdilution assay [14]. Emulsifying agents and organic solvents were excluded when possible, as several authors argued that such additives distort susceptibility tests [12,15]. The trials included a wide variety of EOs, available from three different German essential oil retailers and was focused on the two foodborne pathogenic model bacteria: *Escherichia coli* and *Staphylococcus aureus.*

## 2 Materials and methods

### 2.1 Essential oils

Essential oils were provided by three different German essential oil manufacturers (Neumond GmbH, Raisting, Germany; Frey&Lau GmbH, Henstedt-Ulzburg, Germany; Düllberg Konzentra GmbH, Hamburg, Germany). Overall, essential oils from 86 plant varieties were accessible, whereof 38 equal varieties where available in triplicate and 20 in duplicate, but from the different sources, respectively. Altogether 179 commercial oil samples were tested. The investigated samples did not contain additives or solvents and were confirmed to be natural by the manufacturers. Furthermore, EOs were considered sterile. EO samples were stored in resealable vials at 5 °C in the dark, but were allowed to adjust to room temperature prior to investigation. The samples were sorted in terms of plant name and the respective plant parts they originated from according to the manufacturer information. Geographical origins were excluded as information was mostly not available.

### 2.2 Bacterial strains and growth conditions

The Gram-positive bacterium *Staphylococcus aureus* DSM 1104 and the Gram-negative bacterium *Escherichia coli* DSM 1103 were used as test organisms. The strains were obtained from the Leibniz Institute DSMZ German Collection of Microorganisms and Cell Cultures (DSMZ, Braunschweig, Germany) and are both recommended for antimicrobial susceptibility testing. Stock cultures were grown in 100 ml sterile tryptic soy broth (TSB) (Oxoid, Hampshire, United Kingdom) (pH = 7.2) in shaking flasks at 37 °C for 18 h until their early stationary growth phase. Prior to use, cells were washed twice in sterile ¼*Ringer's solution (Oxoid, Hampshire, United Kingdom) at 9000 rpm for 10 min, respectively. Cell count was adjusted to 1.0*10^8^ cfu/ml in ¼*Ringer's solution by turbidity measurement at 620 nm wavelength, using a McFarland 0.5 standard. The cell counts were preliminarily validated by plate counts. Cells were kept in Ringer's solution for no longer than 15 min before assay medium inoculation.

### 2.3 Essential oil incorporation

Essential oil incorporation was optimized for the execution of a broth microdilution assay for antibacterial susceptibility testing [16]. To avoid the interfering influences of organic solvents or emulsifying agents, a dispersion approach, as first described by Remmal, Bouchikhi [17], was chosen. To increase their viscosity, Mueller-Hinton broth (MHB) (Merck-Millipore, Darmstadt, Germany) and deionized water were spiked with 0.15 % agarose (Merck-Millipore, Darmstadt, Germany) prior to sterilization. All media were adjusted to pH 7.0 ± 0.1. EO stock solutions were adjusted to 12.8 mg/ml in glass vials which were sealed and vigorously shaken for 30 s. Oil containing stock dispersions turned slightly white and were stable towards phase separation for up to 48 h. Air bubbles were driven out by slight vortexing.

A small number of EOs was found to be unstable in dispersion, due to complex-formation and clouding. For these EOs dimethyl sulfoxide (DMSO) was used to aid oil incorporation. Final DMSO concentration was 4.4 % (v/v) and had no growth inhibitory effects. Oils incorporated in DMSO are marked separately in the results tables.

### 2.4 Broth microdilution assay

Antibacterial susceptibility testing was performed according the CLSI laboratory standard for broth microdilution assays [16]. Concentrations tested in the assays ranged from 6400 to 50 μg/ml in bisecting dilution steps. Assays were performed using sterile 96 well microplates (transparent, F-bottom) (Greiner bio-one, Frickenhausen, Germany). Each concentration was tested in triplicate and a single blank (broth with corresponding EO-concentration). Three oils were tested in parallel per plate (n = 3). Ultimately, each well contained 100 μl of EO dispersion. Inoculum was prepared by a hundredfold dilution of the adjusted cell suspension in double concentrated (2*) MHB. Each well was spiked with 100 μl inoculated 2*MHB resulting in a final cell count of 1.0*10^5^ cfu per well. Inoculation media were used immediately to avoid growth dependent shifts in the cell count. Growth, sterility and quality controls were analyzed for every culture, but on separate microplates. Chloramphenicol (2.0–0.008 μg/ml) served as control bacteriostatic to assess cell susceptibility for each batch culture (data not shown). Prior to incubation for 24 h at 37 °C, the inoculated plates were shaken orbital for 30 s on a plate mixer (Kisker, Steinfurt, Germany). To avoid loss of volatile essential oil and humidity during incubation each plate was sealed with a sterile, gas-permeable seal (BREATHseal, Greiner bio-one, Frickenhausen, Germany). Turbidity measurement was performed after incubation in a microplate reader (Tecan, Männedorf, Switzerland) at 595 nm. The seals were removed and plates were shaken orbital for 30 s. Each well was measured at nine spots with 5 flashes per spot. Obtained spot-OD-values of each well were averaged as well-OD-values. Values of parallel wells were averaged afterwards and blank-OD-values were subtracted. The minimal inhibitory concentration (MIC) was defined as the lowest concentration tested which did not allow cell growth within 24 h at 37 °C.

## 3 Results

The MIC values of 179 essential oils from 86 plant species against *E. coli* DSM 1103 and *S. aureus* DSM 1104, determined in a broth microdilution assay, are presented in Table 1. The results show growth inhibitory activities for the majority of the tested EO samples. Inhibition was generally stronger against the Gram-positive bacterium *S. aureus* than against Gram-negative *E. coli.* At or below the preliminary defined critical concentration of 400 mg/ml 46 EO samples from 30 plant genera inhibited *S. aureus,* whereas *E. coli* was only inhibited by 22 samples from 12 plant genera. EO varieties from different providers rarely revealed identical MIC values. Nonetheless, *Azadirachta indica, Backhousia citriodora, Cinnamomum cassia, Cinnamomum verum, Leptospermum scoparium, Litsea cubeba, Nardostachys jatamansi, Origanum vulgare, Pogostemon cablin, Santalum album, Thymus zygis* and *Vetiveria zizanoides* were found to be the most inhibitory EOs against *S. aureus* with MIC values of 50 μg/ml. Only four EOs, *Cinnamomum cassia, Cinnamomum verum, Origanum vulgare* and *Thymus zygis* could exhibit inhibitory effect against *E. coli* at 50 μg/ml. But certain other EOs from *Backhousia citriodora, Cupressus sempevirens, Cymbopogon citratus, Cymbopogon martini, Cymbopogon nardus, Litsea cubeba, Origanum majorana, Origanum vulgare,* and *Syzygium aromaticum* still revealed promising activity with MICs between 100 and 400 μg/ml against *E. coli*.

**Table 1:** Minimal inhibitory concentrations (μg/ml) of essential oils from three different manufacturers (a, b, c) against *Staphylococcus aureus* DSM 1104 and *Escherichia coli* DSM 1103.

	plant botanical name	oil common name	extracted plant part	MIC (μg/ml)					
				*S. aureus*			*E. coli*		
				a	b	c	a	b	c
1	*Abies alba*	silver fir	branches	n. I.			n. I.		
2	*Abies procera*	noble fir	branches		n. I.	n. I.		n. I.	n. I.
3	*Achillea millefolium*	yarrow	herb	6400			n. I.		
4	*Anethum graveolens*	dill	seeds + herb		n. I.			n. I.	
5	*Angelica archangelica*	garden angelica	roots	400	n. I.	n. I.	1600	1600	n. I.
6	*Anthemis nobilis*	roman chamomile	blossom	200*			n. I.*		
7	*Artemisia dracunculus*	tarragon	leaves		n. I.			n. I.	
8	*Artemisia pallens*	davana	herb	6400		n. I.	n. I.		n. I.
9	*Azadirachta indica*	neem	seeds	50*			1600*		
10	*Backhousia citriodora*	lemon myrte	herb	50			200		
11	*Boswellia carterii*	olibanum	resin	1600		n. I.	n. I.		n. I.
12	*Cananga odorata*	ylang-ylang	blossom	n. I.	n. I.	n. I.	n. I.	n. I.	n. I.
13	*Canaricum luzonicum*	elemi	resin	400		n. I.	6400		n. I.
14	*Carum carvi*	caraway	seeds	800	n. I.	3200	3200	6400	1600
15	*Cedrus atlantica*	atlas cedar	wood	n. I.			n. I.		
16	*Cinnammomum camphora*	ravintsara	leaves	3200			6400		
17	*Cinnamomum camphora*	camphor	branches	n. I.		n. I.	6400		6400
18	*Cinnamomum camphora Sieb*	ho	leaves	1600			800		
19	*Cinnamomum cassia*	chinese cinnamon	branches	50	50	50	200	50	50
20	*Cinnamomum verum*	true cinnamon	bark	200	50	800	200	50	200
21	*Cistus ladaniferus*	cistrose	leaves + branches	400			n. I.		
22	*Citrus aurantifolia*	lime	peel	1600	800	3200	6400	800	6400
23	*Citrus aurantium amara*	bitter orange	peel	1600	n. I.	3200	1600	n. I.	1600
24	*Citrus aurantium amara*	neroli	blossom	6400	800	3200	3200	800	6400

	plant botanical name	oil common name	extracted plant part	MIC (μg/ml)					
				*S. aureus*			*E. coli*		
				a	b	c	a	b	c
25	*Citrus aurantium bergamina*	bergamot	peel	6400	6400	n. I.	6400	n. I.	n. I.
26	*Citrus limon*	petit grain	leaves + branches	1600	3200	6400	1600	3200	3200
27	*Citrus medica limonum*	lemon	peel	6400			n. I.		
28	*Citrus paradisi*	grapefruit	peel	800	n. I.	n. I.	3200	n. I.	n. I.
29	*Citrus reticulata*	mandarine	peel	6400	n. I.	n. I.	n. I.	n. I.	n. I.
30	*Citrus sinensi*	orange	all	n. I.	n. I.		3200	3200	
31	*Citrus sinensi*	blood orange	peel	n. I.		n. I.	3200		n. I.
32	*Commiphora myrrha*	myrrh	leaves + branches	100			n. I.		
33	*Coriandrum sativum*	coriander	seeds	1600	1600	1600	800	1600	1600
34	*Corymbia citriodora*	lemon eucalyptus	leaves	800			1600		
35	*Cupressus sempervirens*	cypress	leaves + branches	n. I.	n. I.	n. I.	n. I.	n. I.	n. I.
36	*Cymbopogon citratus*	lemongrass	gras	400	100	800	400	400	800
37	*Cymbopogon martinii*	palmrose	gras	400	200	800	200	200	800
38	*Cymbopogon nardus*	citronella	gras	200			200		
39	*Daucus carotta*	carrot	seeds	n. I.	n. I.	n. I.	n. I.	n. I.	n. I.
40	*Elettaria cardamomum*	cardamom	seeds	800	6400	6400	n. I.	6400	6400
41	*Eucalyptus globulus*	eucalyptus	leaves	400	n. I.	n. I.	6400	n. I.	6400
42	*Eucalyptus radiata*	peppermint	leaves	3200			6400		
43	*Foeniculum vulgare*	fennel	seeds	n. I.	n. I.	n. I.	n. I.	n. I.	n. I.
44	*Helichrysum italicum*	curry plant	buds	1600			1600		
45	*Jasminum grandiflorum*	jasmin	blossom	3200	n. I.		n. I.	n. I.	
46	*Juniperus communis*	common juniper	fruits	n. I.	n. I.	n. I.	n. I.	n. I.	n. I.
47	*Laurus nobilis*	bay laurel	leaves	800		n. I.	800		n. I.
48	*Lavandula angustifolia*	lavender	inflorescence	1600	1600		800	1600	
49	*Lavandula hybrida*	lavender	inflorescence	3100			800		

	plant botanical name	oil common name	extracted plant part	MIC (μg/ml)					
				*S. aureus*			*E. coli*		
				a	b	c	a	b	c
50	*Lavandula spica*	lavender	inflorescence	400			800		
51	*Leptospermum scoparium*	manuka	leaves + branches	50		3200	6400		n. I.
52	*Lippia citriodora*	verbena	herb	400			1600		
53	*Litsea cubeba*	litsea cubeba	fruits	50	100		400	200	
54	*Matricaria chamomilla*	blue chamomile	blossoms	6400*	800*	1600*	n. I.*	n. I.*	n. I.*
55	*Melaleuca alternifolia*	teatree	leaves + branches	800	3200	n. I.	1600	3200	n. I.
56	*Melaleuca cajuputi*	cajeput	blossoms + buds	800	3200	3200	6400	n. I.	3200
57	*Melaleuca quinquenervia*	niaouli	leaves + branches	200	100	3200	6400	n. I.	6400
58	*Melissa officinalis*	lemon balm	blooming herb	100			800		
59	*Mentha arvensis*	field mint	leaves	400		1600	800		1600
60	*Mentha piperita*	peppermint	blooming herb	200	1600	3200	1600	800	1600
61	*Mentha piperita citrata*	bergamot mint	blooming herb	1600			1600		
62	*Mentha spicata*	spearmint	herb	800		6400	800		1600
63	*Myrtus communis*	myrtle	leaves + branches	3200		n. I.	6400		n. I.
64	*Nardostachys jatamansi*	spikenard	roots	50		6400	n. I.		n. I.
65	*Ocimum basilicum*	basil	blooming herb	1600	6400	n. I.	3200	n. I.	n. I.
66	*Origanum majorana*	marjoram	blooming herb	400	800		800	400	
67	*Origanum vulgare*	oregano	herb	100	50	50	50	100	50
68	*Pelargonium graveolens*	rose geranium	leaves	200	1600	1600	800	3200	1600
69	*Picea abies*	norway spruce	leaves + branches	1600	n. I.	800	6400	n. I.	800
70	*Pimpinella anisum*	anise	seeds	n. I.	n. I.	n. I.	n. I.	n. I.	n. I.
71	*Pinus cembra*	swiss pine	needles	n. I.			n. I.		
72	*Pinus mugo*	mountain pine	branches + needles	1600	n. I.	n. I.	n. I.	n. I.	n. I.
73	*Piper nigrum*	pepper	seeds	100	n. I.	n. I.	n. I.	n. I.	n. I.
74	*Pogostemon cablin*	patchouli	leaves	50	200	200	n. I.	n. I.	n. I.

	Plant botanical name	Oil common name	Extracted plant part	MIC (μg/ml)					
				*S. aureus*			*E. coli*		
				a	b	c	a	b	c
75	*Pinus sylvestris*	scots pine	needletips	400	n. I.	n. I.	n. I.	n. I.	n. I.
76	*Rosmarinus officinalis*	rosemary	herb			n. I.			n. I.
77	*Salvia lavandulifolia*	salvia spanish	herb	800	3200	6400	6400	n. I.	n. I.
78	*Salvia officinalis*	salvia	herb	800			1600		
79	*Salvia sclarea*	clary sage	blossoms + leaves	n. I.	3200	n. I.	n. I.	n. I.	n. I.
80	*Santalum album*	indian sandalwood	wood	50*	50*		n. I.*	n. I.*	
81	*Syzygium aromaticum*	clove	buds	100		1600	400		800
82	*Thymus mastichina*	thyme spanish	blooming herb	1600			1600		
83	*Thymus zygis*	thyme linalool	blooming herb	800			400		
84	*Thymus zygis*	thyme thymol	blooming herb	100	50	200	50	50	
85	*Vetiveria zizanoides*	vetiever	roots	800	50	3200	n. I.	n. I.	n. I.
86	*Zingiber officinale*	ginger	roots	800	n. I.	n. I.	1600	n. I.	n. I.

a – Neumond GmbH.

b – Frey&Lau GmbH.

c – Düllberg Konzentra GmbH.

blank space – EO not available.

n. I. – no inhibition.

* - contains DMSO.

Some EOs did not show any antibacterial activities. *Cananga odorata, Cupressus sempervirens, Daucus carotta, Foeniculum vulgare, Juniperus communis, Pimpinella anisum* oils were available from each provider, but did not show any activity against *S. aureus* or *E. coli,* respectively*. Artemisia pallens, Boswellia carterii, Matricaria chamomilla, Pinus mugo, Piper nigrum, Pogostemon cablin, Pinus sylvestris* and *Vetiveria zizanoides* only exhibited inhibitory activity against *S. aureus.* Growth of *E. coli* was not affected by these EOs. For *Pogostemon cablin* and *Vetiveria zizanoides* these findings were particularly distinct, as the three EO samples from the different providers revealed the same results for *E. coli*. Exclusive inhibitory potential against *E. coli* was only shown for samples from *Cinnamomum camphora* and *Citrus sinensis*.

## 4 Discussion

The number of scientific studies on the antimicrobial activity of plant essential oils has strongly grown over the last three decades. Due to the use of many different microbiological methods for susceptibility testing and different definitions of antimicrobial activity, the comparability of studies on essential oils is often critical. Many studies focus on selected EOs, providing insight into their activity against one or more microorganisms [4, 5, 7, 8,18], but only few publications compress information by testing multiplicities of essential oils with a defined single method [2, 3, 13]. Therefore, this study was conducted to investigate as many EOs as possible with a single quantitative microbiological method, to achieve maximum comparability.

As scientifically reviewed elsewhere, thyme, oregano and certain cinnamon EOs, as well as their major constituents, carvacrol, cinnamaldehyde and thymol, are known to possess outstanding inhibitory potential against gram-positive and gram-negative bacteria [3, 10, 13]. Therefore the MICs of thyme and oregano EOs can be set as the benchmark for other EOs. On this basis, it can be asserted that *Azadirachta indica, Backhousia citriodora, Litsea cubeba* and *Nardostachys jatamansi* exhibit comparably high antibacterial activity. These EOs were especially active against *S. aureus*, fully inhibiting its growth at 50 μg/ml (the lowest concentration tested). Furthermore, the EOs from these plants appear to be scientifically less well investigated in terms of antimicrobial activity, as only a small number of preliminary findings exist [19,20,21,22].

*Backhousia citriodora* and *Litsea cubeba* mainly consist of Citral [23,24], a monoterpenoid aroma compound which is generally known to possess strong antimicrobial potential [23,25,26]. *Litsea cubeba* inhibited *S. aureus* at 50 or 100 μg/ml and *E. coli* at 400 or 200 μg/ml, depending on the EO variety tested. In comparison to other essential oils *Litsea cubeba* EO is produced in high amounts and cheaply available. Further research is necessary to identify its full antimicrobial spectrum and to optimize its potential. As the tested *Litsea cubeba* EOs tested in this study fell below the defined food application limit of 400 μg/ml they might be considered a promising candidate for food preservative applications, also due to its unique, refreshing aroma [23,27]. The main active compound of *L. cubeba* EOs is the monoterpene Citral, which has been found to be positive in terms of sensory effects when used as an antimicrobial compound in food products [28].

*Nardostachys jatamansi* and *Azadirachta indica* might also become interesting regarding food preservation applications, as both plants are important in traditional Indian medicine and consequently have histories of safe use [29,30]. *Nardostachys jatamansi* is dominated by different sesquiterpenes which, to our best knowledge, have not been investigated concerning antimicrobial activities yet [30]. The essential oil from *Azadirachta indica*, commonly known as neem-tree essential oil, mainly consists of the compounds Azadirachtin and Nimbin. The compounds are known to possess antimicrobial activity, but are predominately used as spermicides [29,31].

By comparing the results of EOs from a single plant species, but from different manufacturers, it becomes evident that simple postulations regarding the antibacterial effect of a certain EO cannot be made easily. As shown in Table 1, most EO varieties revealed differing MIC values when purchased from another manufacturer. Possible reasons may be versatile, as chemical composition is affected by various external factors, such as geographic origin, environmental conditions, point of harvest or other processing dependent influences [32,33,34]. These findings are in line with the results from previous works [35,36] and enhance the often requested need for chemical characterizations of antimicrobial EOs to identify the active compounds and their interdependencies [3]. Consequently, EO optimization and standardization regarding antimicrobial activity appears to be inevitable for application.

This study also revealed results which differ greatly from those reported by others. The lacking growth inhibition by *Cananga odorata, Cupressus sempervirens, Juniperus communis* or *Pimpinella anisum* may be due to the comparably low concentrations used. Authors, who found these oils to be inhibitory, used way higher concentrations, revealing MICs ranging from 12.5 mg/ml for *Cupressus sempervirens* EO against *E. coli* [37] to 40 %_v/v_ for *Juniperus communis* EO versus *S. aureus* [38]. In regard of future applications in food systems and the strong sensory impact of essential oils on food these EO varieties appear to be unsuitable. Given the focus of application it is apparent to select essential oils with very low MICs. As described before, we defined a critical MIC level of 400 μg/ml in order to identify EO varieties with a greater applicability concerning food preservation. In general it is recommended to couple food application studies with sensory profiling trials. Another peculiarity in this study is the fact, that none of the *Foeniculum vulgare* EOs showed any inhibitory activity. In previous studies fennel seed essential oil was found to be bactericidal at comparable concentrations between 20 μl ml and 80 μl/ml against *E. coli* and *S. aureus* by Dadalioglu and Evrendilek [39]. In this case clarification can only be achieved by chemical analysis of the respective oils which has not been performed as part of our investigations, due to the more broadened approach. On the other hand it was once more affirmed, that *Daucus carotta* essential oils completely lack antimicrobial activity, as also stated by Hammer, Carson [3].

## 5 Conclusion

In summary, this study provides insight into the *in vitro* antibacterial activity of a wide variety of essential oils from many different plant genera against *E. coli* and *S. aureus*. The data contributes to the ongoing scientific investigation regarding the application of essential oils as natural food preservative agents. As the comparison of MICs from different studies is most often difficult, due to the use of varying quantitative or semi-quantitative methods, this study aimed to normalize the discussion by testing a wide variety of plant essential oils with a single, standardized quantitative method for MIC detection. After benchmarking EOs from thyme and oregano as the most active, EO varieties from *Azadirachta indica* and *Litsea cubeba* were identified as promising candidates concerning possible applicability in food.

## Declarations

### Author contribution statement

Julian Thielmann: Conceived and designed the experiments; Performed the experiments; Analyzed and interpreted the data; Contributed reagents, materials, analysis tools or data; Wrote the paper.

Pamina Kazman: Performed the experiments; Analyzed and interpreted the data.

Peter Muranyi: Analyzed and interpreted the data; Wrote the paper.

### Funding statement

This work was partially funded by the Federal Ministry of Economics and Technology (BMWi) via the industrial collective research program of the German Federation of Industrial Research Associations (AiF) under contract number IGF 99EN/1.

### Competing interest statement

The authors declare no conflict of interest.

### Additional information

No additional information is available for this paper.
